# Injury incidence and risk factors in youth soccer players: a systematic literature review. Part I: epidemiological analysis

**DOI:** 10.5114/biolsport.2023.109961

**Published:** 2022-01-03

**Authors:** Mauro Mandorino, António J. Figueiredo, Masar Gjaka, Antonio Tessitore

**Affiliations:** 1 Department of Movement, Human and Health Sciences, University of Rome “Foro Italico”, Rome, Italy; 2 University of Coimbra, Faculty of Sport Sciences and Physical Education, Research Unit for Sport and Physical Activity, Coimbra, Portugal; 3 Department of Sport and Movement Science, University for Business and Technology, Pristina, Republic of Kosovo

**Keywords:** Youth soccer, Injury, Prevention, Severity, Risk factors, Maturity

## Abstract

The analysis of the epidemiological data and the risk factors underlying injuries is crucial to promote prevention strategies in young soccer players. The objective of the present study was to perform a systematic literature review on the epidemiological data, described in the first part, and injury risk factors, presented in the second part. After electronic database searching, articles in line with the inclusion criteria were selected for the systematic review. Epidemiological data were extracted and discussed in this first part of the review. Data were grouped as follows: injury incidence, injury severity, and re-injury, injury types, injury mechanisms, and anatomical location. The principal findings of this first part of the review are as follows: (1) injury incidence is higher in older players and during matches than during training; (2) sex and maturity status may increase risk of injury; (3) male soccer players are more prone to muscle strains and ligament sprains while female players suffer more ligament sprains; (4) most injuries are located in the ankle and thigh in young male soccer players, and in the ankle and knee in female players; (5) severe injuries are less frequent but the incidence increases in older players. Re-injuries represent only a small percentage. Although soccer is considered a safe sport, many injuries are recorded in young soccer players every year. Injury predisposition changes in relation to age, sex, and biological age. Coaches and physical trainers should be aware of individual differences in order to promote prevention strategies and personalised training.

## INTRODUCTION

Football (soccer) is the most popular sport in the world, as witnessed by both the huge TV audience and more than 260 million people actively involved in playing it [1], also encouraged by the health benefits obtained by regular recreational practice. In fact, recreational soccer has been demonstrated to have positive effects on cardiovascular function, body composition, and neuromuscular fitness [2–5]. Moreover, according to the 2016 FIFA report, more than half of the 38 million players officially registered belong to the youth category under 18 years. Although youth soccer seems to be a healthy and relatively safe sport [6–8], adolescent players are constantly exposed to risks of injury. Indeed, soccer is a contact sport characterised by high-intensity activities such as sprints, jumps, and changes of direction [9] that could raise the players’ predisposition to injury. Furthermore, financial rewards and the signing of a professional contract may contribute to increasing the state of stress and anxiety of youth players [10].

Trauma in youth athletes could produce various side effects, such as dropout [11], alteration in the talent development process, long-term sequelae [12], and an economic impact on the health care system [13]. Therefore, understanding the epidemiological data and risk factors underpinning the injury mechanism is crucial. Acquiring such awareness requires a complex analysis due to the numerous elements which may determine the occurrence of an injury. In the sports science literature, many risk factors linked to injuries are commonly categorised in extrinsic (e.g. training load, rules, playing surface) and intrinsic (e.g. flexibility, strength, age, sex, previous injury) factors [14].

Moreover, unlike adults, during biological maturation young athletes experience a time of their life characterised by rapid changes in hormonal release, body size, shape, composition [15], and neuromuscular control [16]. All these factors make young soccer players highly predisposed to the risk of injury. Thus, the analysis of epidemiological data and injury risk factors of youth soccer players is highly needed in order to promote effective prevention strategies. Indeed, according to “the sequence of prevention” introduced by Van Mechelen et al. [17], before applying preventive measures, it is needed to analyse sports injuries (e.g. incidence, severity) and to recognise the underlying risk factors. Therefore, the current systematic literature review aimed to improve this knowledge, providing adequate information to practitioners in order to implement robust preventive strategies.

To date, many epidemiological studies have been carried out in a young soccer population, although the use of different injury definitions, age of samples involved, competition level, and length of follow-up, makes the interpretation of these results difficult. Among the total amount of epidemiological studies, some of these have focused on injury incidence, type, anatomical distribution, or severity [18–22], while others have investigated the risk factors [23–27].

Moreover, several authors have tried to review data on injuries in youth soccer players, but some of these studies are dated [28] or exclusively focused on descriptive epidemiological data [29, 30]. Then, only information about injury incidence and distribution is not enough to understand the suitable prevention strategy in young soccer.

Based on our knowledge, to date, there are no systematic literature reviews that combine epidemiological data with injury risk factors. Therefore, the purpose of the present review is to summarise the evidence related to injury incidence in young soccer players and to match it with the risk factors, in order to understand the mechanism underlying a higher injury predisposition, promote prevention strategies and minimise lost playing time. The present review is organised in two different parts:

– Part 1: epidemiological data review of the injuries in young soccer players.
– Part 2: analysis of the injury risk factors in young soccer players.

## MATERIALS AND METHODS

### Search strategy

A systematic review of the literature was conducted according to the Preferred Reporting Items for Systematic Reviews and Meta-Analyses (PRISMA) statement [31]. The whole research (composed of two parts) aimed at identifying studies concerning injury epidemiological data and injury risk factors in youth soccer players. The eligible studies were searched by two independent researchers consulting the following electronic databases: ERIC (Educational Resources Information Center), PubMed/NCBI (National Center for Biotechnology Information, U.S. National Library of Medicine), Scopus, SPORTDiscus via EBSCOhost and Web of Science (WOS), from inception to October 2019. In each database, the search was performed as follows: [soccer OR football] AND [youth OR young OR adolescen*] AND [injur* OR risk of injury OR impairments].

All the articles were collected using Excel Software (Microsoft Excel 2016, Microsoft Corporation, Washington, USA) to manage duplicates and screening procedures.

### Inclusion and exclusion criteria

The systematic literature review focused on two main topics: injury epidemiological data and injury risk factors in youth soccer players; thus, the inclusion criteria were general and specific for each topic.

General inclusion criteria: (1) published original data (i.e., abstracts, books, reviews, systematic reviews, and meta-analyses were excluded); (2) published in the English language; (3) published in a peer-review scientific journal; (4) articles found on the electronic database up to the 28^th^ of February 2021. Finally, to allow the identification of relevant papers not found during the electronic search, the snowballing technique was applied.

Inclusion criteria for injury epidemiological data: (1) samples of young male and female soccer players (7–18 years old); (2) articles which collected at least one outcome related to injury epidemio-logical data: injury incidence, injury type, severity, re-injury, anatomical location (3) prospective or retrospective studies.

Inclusion criteria for injury risk factors: (1) samples of young male and female soccer players (5–18 years old); (2) articles that analysed risk factors connected to the onset of injury (3) articles identifying injury predisposition factors (4) prospective, retrospective, cross-sectional studies, randomised control trials (RCT).

**FIG. 1:**
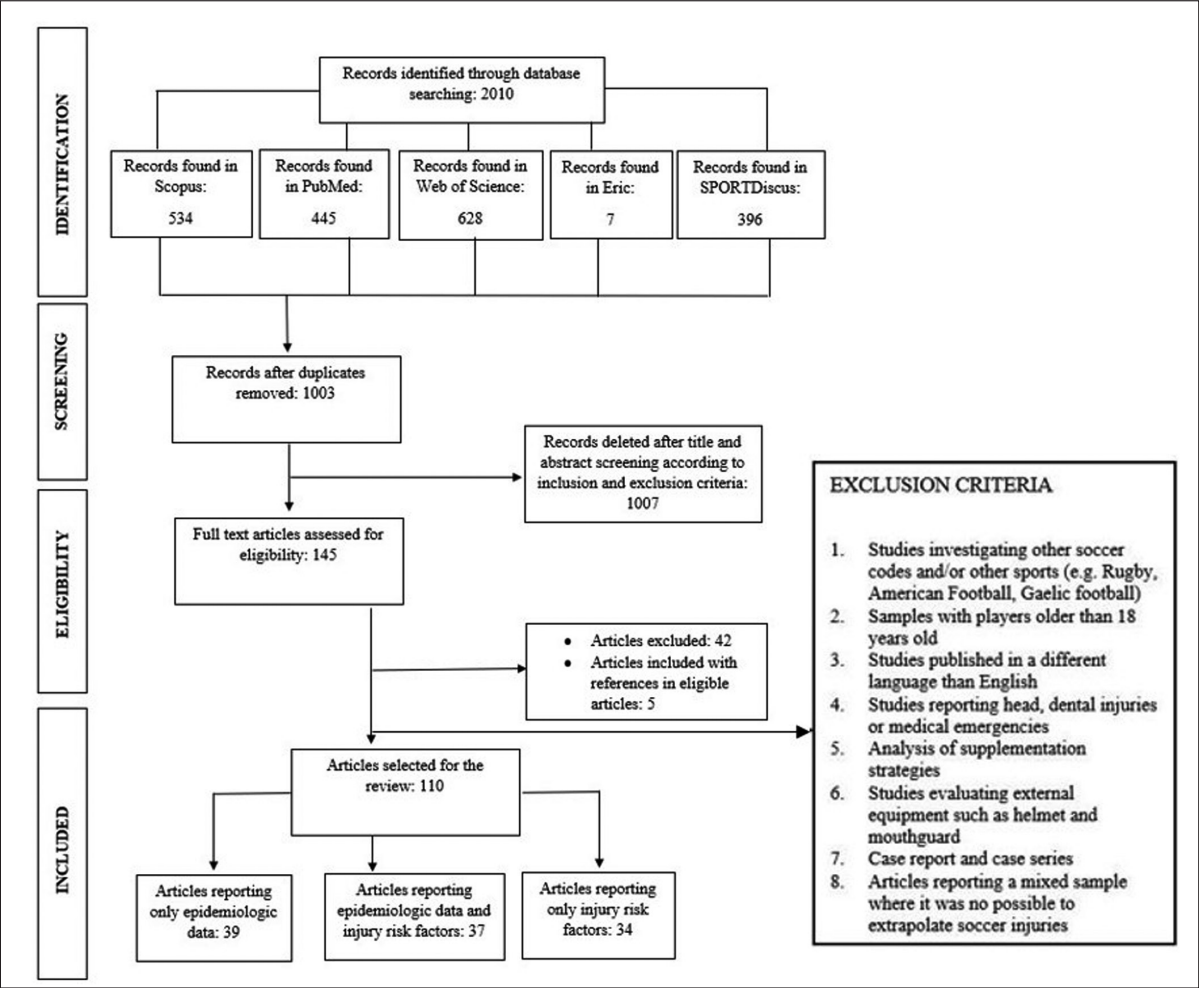
PRISMA Flow Chart.

Exclusion criteria are presented in Figure 1.

### Study selection process

Electronic database searching was initially performed by one reviewer (MM). Then, removal of duplicates was done by two reviewers (MM and AT). After this step, considering the high amounts of articles identified, a preliminary title screening was conducted, and the selected articles were subjected to abstract screening according to the inclusion criteria previously mentioned.

The full text of the articles identified for eligibility were analysed by three reviewers (AT, MG, MM) for the two main topics: injury epidemiological data and injury risk factors. Thus, the included articles were organised separately according to the area of interest and subjected to a data extraction process conducted by two reviewers (AT, MM).

### Article quality assessment

As highlighted in a previous systematic review [32], Delphi [33] or PEDro (Physiotherapy Evidence Database) [34] scales, which are commonly used to assess article quality, present criteria that are not relevant for specific studies, as in the current review. Following the same procedure reported by Hume et al. [32], two authors (MM, AT) independently assessed each article reported in the current review using a 6-item custom methodological quality assessment scale. The six items were (P1) study design (0 = retrospective cohort study, 1 = prospective cohort study); (P2) injury definition (0 = not reported, 1 = reported); (P3) injury severity (0 = not reported, 1 = reported); (P4) sample size (0 = less than 20 subjects recruited, 1 = more than 20 subjects recruited); (P5) participants’ level (0 = non-elite, 1 = elite/sub-elite); (P6) follow-up period (0 = less than six months, 1 = more than six months). The evaluation process together with the final quality score are presented in Table 1. The quality score calculated was not considered as an exclusion criterion.

**TABLE 1:** General information of studies selected.

REFERENCES	STUDY DESIGN(QUALITY SCORE)	P1	P2	P3	P4	P5	P6	COUNTRY	DURATION OF DATACOLLECTION	LEVEL OF YOUNGPLAYERS	SEX OF PLAYERS
Andreasen et al. [55]	Prospective injury report (2)	1	0	0	1	0	0	Denmark	5-day tournament	Non-elite	Male/Female
Aoki et al. [26]	Prospective cohort study (4)	1	1	0	1	0	1	Japan	1 season	Non-elite	Male
Bacon and Mauger [80]	Prospective cohort study (5)	1	0	1	1	1	1	UK	2 seasons	Elite	Male
Bianco et al. [67]	Prospective cohort study (6)	1	1	1	1	1	1	Italy	1 season	Elite	Male
Blazkiewicz et al. [38]	Retrospective, cross sectional study(3)	0	1	0	1	0	1	Poland	Not available	Non-elite	Male
Bowen et al. [23]	Prospective cohort study (6)	1	1	1	1	1	1	UK	2 seasons	Elite	Male
Brink et al. [8]	Prospective cohort study (5)	1	1	0	1	1	1	The Netherlands	2 seasons	Elite	Male
Brito et al. [22]	Prospective cohort study (4)	1	1	0	1	0	1	Portugal	1 season	Non-elite	Male
Brito et al. [46]	Prospective cohort study (5)	1	1	1	1	1	0	Portugal	Preseason (6 weeks)	Sub-elite	Male
Bult et al. [27]	Prospective cohort study (6)	1	1	1	1	1	1	The Netherland	3 seasons	Elite	Male
Cezarino et al. [64]	Prospective cohort study (6)	1	1	1	1	1	1	Brazil	1 season	Elite	Male
Clausen et al. [87]	Prospective cohort study (4)	1	1	0	1	0	1	Denmark	1 season	Non-elite	Female
Cloke et al. [95]	Prospective cohort study (5)	1	1	0	1	1	1	UK	6 seasons	Elite	Male
Cloke et al. [37]	Prospective cohort study (5)	1	1	0	1	1	1	UK	3 seasons	Elite	Male
De Ridder et al. [74]	Prospective cohort study (5)	1	1	0	1	1	1	Belgium	3 seasons	Elite	Male
Deehan et al. [18]	Prospective cohort study (5)	1	1	0	1	1	1	UK	5 seasons	Elite	Male
Del Coso et al. [45]	Retrospective cohort study (3)	0	1	0	1	0	1	Spain	1 season	Non-elite/National	Female
Elias [35]	Prospective injury report (4)	1	1	0	1	0	1	USA	10 years (tournament)	Non-elite	Male/Female
Emery & Meeuwisse [84]	Prospective cohort study (3)	1	1	0	1	0	0	Canada	20 weeks	Non-elite	Male/Female
Emery et al. [72]	Prospective cohort study (4)	1	1	1	1	0	0	Canada	13 weeks	Non-elite	Male/Female
Ergun et al. [43]	Prospective cohort study (6)	1	1	1	1	1	1	Turkey	3 seasons	Elite	Male
Frisch et al. [70]	Prospective cohort study (5)	1	1	1	1	0	1	Luxemburg	1 season	Non-elite	Male
Froholdt et al. [78]	Prospective cohort study (5)	1	1	1	1	0	1	Norway	1 season	Non-elite	Male/Female
Hägglund & Waldén [82]	Prospective cohort study (4)	1	1	0	1	0	1	Sweden	1 season	Non-elite	Female
Herdy et al. [52]	Descriptive cross-sectional and correlationalstudy (4)	0	1	0	1	1	1	Brazil	11 months	Elite	Male
Hoff & Martin [79]	Retrospective survey (3)	0	1	0	1	0	1	USA	Not available	Non-elite	Male/Female
Jacobs & Van Den Berg [53]	Retrospective cohort study (4)	0	0	1	1	1	1	Africa	Not available	Elite	Male
Johnson et al. [77]	Prospective cohort study (4)	1	0	0	1	1	1	UK	6 season	Elite	Male
Johnson et al. [73]	Prospective cohort study (5)	1	1	0	1	1	1	UK	2 season	Elite	Male
Kakavelakis et al. [56]	Prospective cohort study (5)	1	1	1	1	0	1	Greece	1 season	Non-elite	Male
Kemper et al. [69]	Prospective cohort study (5)	1	1	0	1	1	1	The Netherland	1 season	Elite/Non-elite	Male
Khodaee et al. [51]	Descriptive epidemiological study (4)	1	1	0	1	0	1	USA	9 seasons	Non-elite	Male/Female
Kofotolis [68]	Prospective cohort study (4)	1	1	0	1	0	1	Greece	1 season	Non-elite	Male
Kolstrup et al. [7]	Prospective cohort study (5)	1	1	0	1	1	1	Denmark	3 seasons	Elite	Male/Female
Kucera et al. [76]	Prospective cohort study (4)	1	1	0	1	0	1	USA	4 seasons	Non-elite	Male/Female
Kuzuhara et al. [21]	Prospective cohort study (4)	1	1	0	1	0	1	Japan	1 season	Non-elite	Male
Le Gall et al. [42]	Prospective cohort study (6)	1	1	1	1	1	1	France	10 seasons	Elite	Male
Le Gall et al. [71]	Prospective cohort study (6)	1	1	1	1	1	1	France	10 seasons	Elite	Male
Light et al. [63]	Prospective cohort study (6)	1	1	1	1	1	1	UK	4 seasons	Elite	Male
Lislevand et al. [49]	Prospective cohort study (3)	1	1	0	1	0	0	Norway	2-day tournament	Non-elite	Female
Maehlum et al. [41]	Prospective cohort study (2)	1	0	0	1	0	0	Norway	6-day tournament	Non-elite	Male/Female
Materne et al. [62]	Prospective cohort study (6)	1	1	1	1	1	1	Qatar	4 seasons	Elite	Male
Materne et al. [97]	Prospective cohort study (6)	1	1	1	1	1	1	Qatar	4 seasons	Elite	Male
McCarroll et al. [54]	Prospective cohort study (2)	1	0	0	1	0	0	USA	4 months	Non-elite	Male/Female
Nagle et al. [92]	Prospective cohort study (5)	1	1	1	1	0	1	USA	8 seasons	Non-elite	Male/Female
Namazi et al. [91]	Prospective cohort study (5)	1	1	0	1	1	1	Iran	1 season	Elite	Male
Nilsson et al. [48]	Prospective cohort study (6)	1	1	1	1	1	1	Sweden	2 seasons	Elite	Male
Nogueira et al. [44]	Prospective cohort study (5)	1	1	1	1	0	1	Portugal	6 months	Non-elite	Male
O’Kane et al. [88]	Prospective cohort study (5)	1	1	0	1	1	1	USA	2 seasons	Elite	Female
O’Kane et al. [116]	Prospective cohort study (5)	1	1	0	1	1	1	USA	2 seasons	Elite	Female
O’Kane et al. [85]	Prospective cohort study (5)	1	1	0	1	1	1	USA	2 seasons	Elite	Female
Olumide & Ajide [19]	Prospective cohort study (4)	1	1	1	1	0	0	Nigeria	3-day tournament	Non-elite	Male
Price et al. [96]	Prospective cohort study (5)	1	1	1	1	0	1	UK	2 seasons	Non-elite	Male
Raya-González et al.[66]	Prospective cohort study (6)	1	1	1	1	1	1	Spain	4 seasons	Elite	Male
Raya-González et al.[50]	Prospective cohort study (6)	1	1	1	1	1	1	Spain	1 season	Elite	Male
Raya-González et al.[25]	Prospective cohort study (5)	1	1	0	1	1	1	Spain	1 season	Elite	Male
Read et al. [36]	Prospective cohort study (6)	1	1	1	1	1	1	UK	1 season	Elite	Male
Renshaw & Goodwin [47]	Prospective cohort study (6)	1	1	1	1	1	1	UK	1 season	Elite	Male
Rosenbaum et al.[93]	Prospective cohort study (3)	1	1	0	1	0	0	USA	2-day tournament	Non-elite	Male/Female
Rössler et al.[59]	Prospective cohort study (5)	1	1	1	1	0	1	Czech Republic and Switzerland	2 seasons	Non-elite	Male/Female
Rössler et al. [83]	Prospective cohort study (5)	1	1	1	1	0	1	Czech Republic and Switzerland	2 seasons	Non-elite	Male/Female
Schiff et al. [58]	Prospective cohort study (4)	1	1	0	1	0	1	USA	1 season	Non-elite	Female
Schiff [57]	Cross-sectional survey (3)	0	1	0	1	0	1	USA	Not available	Non-elite	Female
Schmidt-Olsen et al. [20]	Prospective cohort study (5)	1	1	0	1	1	1	Denmark	1 season	Elite	Male
Sieland et al. [94]	Prospective cohort study (6)	1	1	1	1	1	1	Germany	2 seasons	Elite	Male
Sokka et al. [65]	Prospective cohort study (4)	1	1	1	1	0	0	Finland	20 weeks	Non-elite	Male/Female
Soligard et al. [24]	Prospective cohort study (4)	1	1	0	1	0	1	Norway	1 season	Non-elite	Female
Steffen et al. [86]	Prospective cohort study (4)	1	1	0	1	0	1	Norway	1 season	Non-elite	Female
Sullivan et al. [40]	Prospective cohort study (4)	1	1	0	1	0	1	USA	Not available	Non-elite	Male/Female
Timpka et al. [12]	Prospective cohort study (4)	1	1	0	1	0	1	Sweden	1 season	Non-elite	Male
Tourny et al. [39]	Prospective cohort study (4)	1	0	0	1	1	1	France	3 seasons	Elite	Male
Van der Sluis et al. [89]	Prospective cohort study (5)	1	1	0	1	1	1	The Netherlands	3 seasons	Elite	Male
Van der Sluis et al. [75]	Prospective cohort study (5)	1	1	0	1	1	1	The Netherland	3 seasons	Elite	Male
Volpi et al. [60]	Prospective cohort study (5)	1	1	0	1	1	1	Italy	4 years	Elite	Male
Watson et al. [81]	Prospective cohort study (3)	1	1	0	1	0	0	USA	20 weeks	Non-elite	Female
Watson et al. [90]	Prospective cohort study (3)	1	1	0	1	0	0	USA	20 weeks	Non-elite	Female
Wik et al. [61]	Prospective cohort study (6)	1	1	1	1	1	1	Qatar	4 seasons	Elite	Male

## RESULTS

### Search results

The articles’ selection process is illustrated in the Prisma Flow Chart (Figure1). A total of 110 articles were included in the present systematic literature review in accordance with inclusion and exclusion criteria. Of the 110 articles included, thirty-nine reported only epidemiological data [7, 12, 18–22, 35–66] and thirty-seven combined epidemiological data and injury risk factors [8, 23–27, 67–97]. The remaining articles, reporting only injury risk factors, are discussed in the second part of the current systematic review.

The main findings extracted about epidemiological data and presented in this part 1 have been organised based on the following parameters: injury incidence, injury severity and re-injury, injury types, injury mechanisms, and anatomical location. General information of the studies, including the article quality assessment score, is presented in Table 1.

### Epidemiological data

### Injury definition and collection process

The studies included in the review were characterised by different injury definitions. Thirty-six articles were based on Fuller et al.’s [98] consensus statement published in 2006 [7, 8, 12, 19, 22–24, 26, 27, 43–49, 58, 59, 61–65, 67, 69, 75, 78, 82, 86–91, 93, 97]. Regarding the time loss from soccer activity, eight studies used more than 48 h [18, 36, 37, 42, 71, 74, 95, 96], four studies up to 24 h [51, 56, 57, 92] and only one study used a period of four weeks [96]. Three studies followed Hägglund et al.’s [99] indications [25, 50, 66]. Ten studies defined overall injury as inability to take part in training or competition [21, 38, 40, 68, 70, 72, 73, 76, 79, 84]. Two articles used an acute or overuse definition [85, 90]. The remaining studies did not report [39, 41, 53–55, 77, 80] or used a different injury definition [35, 52].

Moreover, different injury collection strategies were adopted. Most of the studies relied on physiotherapists/medical staff [7, 8, 18, 22, 23, 25, 27, 35–37, 39, 41–49, 52, 55, 60–64, 67–69, 71–75, 78, 80, 82, 89, 91, 93–97, 100]; others, instead, relied on the coaching staff [12, 21, 22, 24, 26, 40, 44, 50, 54, 58, 59, 66, 86, 87, 92, 93], the research team [19, 70, 90] or a self-reported questionnaire/web system/phone interview [38, 51, 53, 56, 57, 65, 76, 79, 83–85, 88, 90]. Two studies did not report any information [20, 77].

### Injury incidence

The injury incidences reported below were calculated as the ratio between the number of injuries and hours of playing exposure per 1000 h. In total, fifty-five articles [7, 8, 12, 18–23, 25–27, 35–37, 39–44, 46–48, 50–53, 56, 61, 63–78, 80, 83, 84, 89, 91–95] reported injury incidence in young male soccer players. The authors identified an overall injury incidence per 1000 h ranging from 0.51 [40] to 18.4 [43]. Specifically, the injury incidence ranged from 2.84 [8] to 47.7 [43] during matches and from 0.9 [22] to 11.14 [8] during training. Three studies, rather than injury incidence, reported injury prevalence, as shown in Table 2 [18, 47, 53]. Moreover, the injury incidence according to chronological age is also reported in Table 2.

**TABLE 2:** Injury incidence summary in young soccer players.

References	Category (participation level)	Maturity	Number of players	Number of Injuries	Average Injury per player (player/n injury)	Injury incidence per 1000 h (prevalence %)			
						Training	Matches	Tournament	Overall
**Male**									
Elias [35]	U12-U19 (Non-elite)		89500	2353				13.39	
	U12			298				11.22	
	U14			606				11.81	
	U16			789				16.05	

Wik et al. [61]	U13-U18 (Elite)		591	1111	1.9	8.2	32.0		12.0
	U13		102	133		6.0	21.2		7.8
	U14		106	164		6.3	23.4		8.5
	U15 U16		117	194		7.4	27.8		10.9
	U17		102	215		8.8	35.9		13.7
	U18		92	234		11.0	43.8		17.0
			72	171		13.2	40.0		18.6
	U19			625					13.46

Light et al. [63]	U9-U21 (Elite)		190	603					2.4
	U9								1.8
	U10								2
	U11								0.7
	U12								1.1
	U13								3
	U14								2.9
	U15								2.5
	U16								2.3
	U18								2.9
	U21								4.8

Materne et al. [97]	U13-U19		454	1565		736 (47.1%)	829 (52.9%)		
		Mature	94	395		209 (52.9%)	186 (47.1%)		
		Early maturers	192	692		300 (43.4%)	392 (56.6%)		
		Normal maturers	158	446		205 (46%)	241 (54%)		
		Late maturers	10	32		22 (68.8%)	10 (31.3%)		

Bianco et al. [67]	U13-U20 (Elite)		80	107		1.15	2.84		1.28
	U13-U16		54	72		1.16	2.20		1.22
	U17-U20		23	35		1.13	4.30		1.40

Bowen et al. [23]	U18-U21 (Elite)		32	138		7.9	33.5		12.1

Deehan et al. [18]	U9-U19 (Elite)		210	685	0.6	334 (49%)	351 (41%)		

Read et al. [36]	U11-U18 (Elite)		609	804					1.32
	U11		83	53					0.64
	U12		88	96					1.09
	U13		83	102					1.23
	U14		90	97					1.08
	U15		71	111					1.56
	U16		86	116					1.35
	U18		107	229					2.14

Cloke et al. [37] ^a^	U9-U18 (Elite)		419	56		0.077	0.862		0.342

Cezarino et al. [64]	U11-U20 (Elite)		228	187		1.41	8.17		1.86
	U11		23	2		0.22	2.72		0.41
	U12		22	8		2.05	NA		1.80
	U13		25	6		0.40	5.47		0.74
	U14		28	21		1.37	9.09		1.64
	U15		28	12		0.81	2.65		0.91
	U16		25	27		2.05	4.58		2.18
	U17		28	46		2.28	13.66		3.05
	U18		16	18		1.42	8.08		1.74
	U20		33	47		1.32	22.48		2.46

Sokka et al. [65]	U9-U14 (Non-elute)		567	321		3.63	24.67		6.29

Kofotolis et al.[68] ^a^	U9-U15 (Non-elite)		677	38					0.38

Kemper et al. [69]	U12-U19 (Elite/Non-elite)		101	134		3.3	18.2		5.9

Frisch et al. [70]	U15-U19 (Non-elite)		67	163		7.1	23.5		10.4

Le Gall et al. [71]	U14 (Elite)		233	588	2.5	4.7	11.8		5.6
		Early maturers	57	146	2.6	4.5	13.2		5.7
		Normal maturers	148	384	2.6	4.8	12.3		5.8
		Late maturers	28	58	2.1	4.3	6.5		4.6

Timpka et al. [12]	U14-U17			44					2.4
	U14 (Non-elite)			9					1.7
	U15 (Non-elite)			0					0
	U15 (elite)			16					6.8
	U16 (Non-elite)			4					1.5
	U16 (Elite)			9					2.8
	U17 (Elite)			6					1.9

Błażkiewicz et al. [38] ^b^	U12-U18 (Non-elite)		33	52					6.48

Raya-González et al. [25] ^c^	U19 (Elite)		22	27		3.87	14.35		5.11

Tourny et al. [39]	U12-U20 (Elite)		412	596					
	U12		38			1.0	9.4		1.5
	U13		53			2.2	16.5		2.8
	U14		57			2.3	28.7		4.1
	U15		51			2.8	36.7		5.0
	U16		52			2.2	29.4		3.7
	U17		52			3.6	24.1		4.8
	U19		51			2.8	30.3		4.4
	U20		58			3.8	42.2		5.7

Sullivan et al. [40]	U8-U19 (Non-elite)		931	19					0.51

Emery et al. [72]	U14-U18 (Non-elite)		317	39					5.55
	U14			16					7.88
	U16			16					5.68
	U18			7					3.22

Maehlum et al. [41]	U12-U18 (Non-elite)			266				9.9	
	U12							9.3	
	U13							9.1	
	U16							11.2	
	U18							8.6	

Johnson et al. [73]	U11-U16 (Elite)		76	88			15.8		
	U11		24	6			7.3		
	U12		21	12			13.4		
	U13		22	16			17.1		
	U14		15	23			22.2		
	U15		16	20			16.0		
	U16		14	11			17.0		
		Pre-PHV					11.5		
		Circa-PHV					24.5		
		Post-PHV					16.4		
		Early maturers					Not reported		
		Normal maturers					18.5		
		Late maturers					6.4		

De Ridder et al. [74]	U11-U17 (Elite)		133	68					2.0

Olumide & Ajide [19]	U11-U19 (Non-elite)		90	15				113.6	

Le Gall et al. [42]	U14-U16 (Elite)		660	1152	2.2	3.9	11.2		4.8
	U14		240	420	2.2	4.1	9.5		4.9
	U15		220	361	2.1	3.7	10.4		4.6
	U16		200	371	2.3	3.8	14.2		5.2

Aoki et al. [26]	U14-U16 (Non-elite)		301	425					4.04

Schmidt-Olsen et al. [20]	U13 (Elite)		247	137	0.55				3.4
	U15		112	67	0.60				3.8
	U17		137	108	0.79				4.0

Kolstrup et al. [7]	U12-U19 (Elite)		32380	1091				13.1	

Ergün et al. [43]	U17-U19 (Elite)		24	44		10.5	47.7		18.4
	U17		24	25		14.9	35.9		19.4
	U18		24	5		7.6	45.5		15.2
	U19		24	14		4.8	74.1		18.1

Kuzuhara et al. [21]	U10-U12 (Non-elite)		89	29		1.49	6.43		2.59

Nogueira et al. [44]	U17-U19 (Non-elite)		529	248		2.06	14.22		3.87
	U17		290	138		2.14	12.60		3.74
	U19		239	110		1.97	16.01		4.02

Brito et al. [22]	U13-U19 (Non-elite)		674	199		0.9	4.7		1.2
	U13		179			0.5	2.0		0.6
	U15		169			0.7	6.1		1.1
	U17		165			1.1	3.7		1.4
	U19		161			1.2	7.1		1.7

Brito et al. [46] ^d^	U13-U19 (Sub-elite)		912	53		1.8	6.8		2.5
	U13					2.0	1.9		1.9
	U15					2.3	6.3		2.7
	U17					1.4	11.0		2.8
	U19					1.7	7.2		2.4

Kucera et al. [76]	U12-U18 (Non-elite)		928	467					4.3

Renshaw & Goodwin [47]	U9-U18 (Elite)		181	127		64 (50%)	41 (32%)		

Nilsson et al. [48]	U15-U19 (Elite)		43	61	0.7	5.6	15.5		6.8

Bult et al. [27]	U12-U19 (Elite)		170	620	2.0				8.34
	U12		17		1.2				5.86
	U13		50		1.0				5.12
	U14		54		1.6				7.41
	U15		54		2.6				12.44
	U16		53		2.1				8.65
	U17		38		3.1				10.12
	U19		43		2.2				6.90
		Pre-PHV							6.99
		Circa-PHV							9.56
		Post-PHV							8.66

Johnson et al. [77]	U9-U16 (Elite)		292	476		1.44	10.5		2.23
		Early Maturers							1.8
		Normal Maturers							1.5
		Late Maturers							1.4

Froholdt et al. [78] ^e^	U6-U16 (Non-elite)		1260	115		0.5	5.4		2.2
	U6-U12		870	44					1.3
	U13-U16		390	71					2.1

Brink et al. [8]	U15-U18 (Elite)		53	320		11.14	37.55		

Raya-González et al. [50] ^f^	U14-U19 (Elite)		118	38		0.72	5.63		1.23
	U14		39	8		0.51	4.14		0.91
	U16		39	12		0.48	7.13		1.28
	U19		40	18		1.04	5.05		1.41

Raya-González et al. [66]	U14-U19 (Elite)		309	464		2.10	10.16		2.93
	U14			84		1.95	6.01		2.39
	U16			111		1.88	9.12		2.75
	U19			142		2.07	11.01		2.86

Khodaee et al. [51]	U14-U18 (Non-elite)			2912		1.04	3.68		1.83

Herdy et al. [52]	U11-U20 (Elite)		143	200	1.40				
	U11		30	12	0.40				
	U13		34	15	0.44				
	U15		23	46	2.00				
	U17		24	66	2.75				
	U20		32	61	1.91				

Bacon & Mauger [80]	U18-U21 (Elite)		41	85		3.72	5.84		

Jacobs & Van den Berg [53]	U14-U18 (Elite)		169	544		297 (55%)	247 (45%)		

Rössler et al. [83] ^h^	U8-U12 (Non-elite)		6038	417		0.61	4.57		

Emery & Meeuwisse [84]			317	113					5.18

Kakavelakis et al. [56]	U12-U15 (Non-elite)		287	209		3.3	5.6		4.0

Rössler et al. [59] ^h^	U8-U12 (Non-elite)		6038	417		0.61	4.57		1.05
	U8		1770	56		0.30	2.49		0.51
	U10		2247	115		0.41	3.59		0.77
	U12		2021	246		1.07	6.14		1.78

Van der Sluis et al. [89]	(Elite)	Pre-PHV				2.57	12.49		
		Circa-PHV				4.19	20.50		
		Post-PHV				3.84	23.08		

Namazi et al. [91]	U18-U21 (Elite)		73	22					2.1

Cloke et al. [95] ^a^	U9-U16 (Elite)		14776	2563		1344 (52.4%)	1121 (43.7%)		

Nagle et al. [92]	U14-U18 (Non-elite)			2110		0.58	1.80		0.95

Rosenbaum et al. [93]	U10-U15 (Non-elite)			26				7.26	

**Female**									
Elias [35]	U12-U19 (Non-elite)		89500	1387				14.78	
	U12			191				12.64	
	U14			439				16.92	
	U16			511				17.68	
	U19			246				10.64	

Soligard et al. [24]	U16 (Non-elite)		202	259		89 (35.5%)	167 (64.5%)		

Sullivan et al. [40]	U8-U19 (Non-elite)		341	15					1.1

Emery et al. [72]	U14-U18 (Non-elite)		317	39					5.62
	U14			20					7.92
	U16			14					5.74
	U18			5					2.53

Maehlum et al. [41]	U14-U18 (Non-elite)			145				17.6	
	U14							13.0	
	U16							20.5	
	U18							15.9	

Kolstrup et al. [7]	U12-U19 (Elite)		13226	740				20.3	

Del Coso et al. [45]	U18 (Elite/Non-elite)		12540	904	0.072				

Kucera et al. [76]	U12-U18 (Non-elite)		555	320					5.3

Lislevand et al. [49]	U13-O16 (Non-elite)		938	123				93.3	
	U13		433	50				116.0	
	U16		213	47				116.6	
	O16		292	26				53.7	

Froholdt et al. [78] ^e^	U6-U16 (Non-elite)		619	38		0.4	4.6		2.0
	U6-U12		350	11					1.0
	U13-U16		269	27					1.6
Khodaee et al. [51]	U14-U18 (Non-elite)			3242		1.07	5.25		2.33

Watson et al. [81]	U13-U18 (Non-elite)		54	28					5.3

Hägglund & Waldén [82] ^g^	U14-U18 (Non-elite)		4556	96		0.074	1.09		0.35

O’Kane et al. [85] ^i^	U12-U15 (Elite)		351	83					1.9

Clausen et al. [87] ^g^	U15-U18 (Non-elite)		380	34					1.8

Schiff [57]	U11-U14 (Non-elite)		103	44		1.0	6.1		2.2

Nagle et al. [92]	U14-U18 (Non-elite)			2639		0.74	2.88		1.39

Rosenbaum et al. [93]	U11-U18 (Non-elite)			42				7.55	

Sokka et al. [65]	U9-U14 (Non-elite)		163	89		3.47	30.59		7.20

Note: Prevalence was reported in parenthesis (%) preceded by the absolute number

Four studies [71, 73, 75, 77] reported injury incidence according to players’ biological age and three [27, 73, 89] according to peak height velocity (PHV). Instead, one study adopted the Khamis-Roche equation [63]. Five studies [7, 19, 35, 41, 93] recorded injury incidence during tournaments with an injury rate ranging from 7.26 [93] to 113.6 [19].

In the articles reporting female injuries [7, 24, 35, 40, 41, 45, 49, 51, 57, 65, 72, 76, 78, 82, 85, 87, 90, 92, 93] the overall injury incidence ranged between 1.1 [40] and 7.20 [65]. During training the value varied from 0.74 [92] to 3.47 [65], whereas during matches it varied from 2.88 [92] to 30.59 [65]. Four studies [7, 35, 41, 49] recorded injury data during a female tournament.

Injury incidence for specific cases (e.g. ankle injuries, non-contact injuries, traumatic injuries) is presented in Table 2.

### Injury incidence according to participants’ level

Epidemiological information was also extracted and discussed according to participants’ level. In elite young male soccer players, an overall injury incidence ranging from 1.23 [50] to 12.1 [23] was reported. In non-elite young male soccer players, the range was from 0.51 [40] to 10.4 [70]. Specifically, a training injury rate from 0.72 [50] to 11.14 [8] in elite young soccer players, and from 0.58 [92] to 7.1 [70] in non-elite young soccer players were identified. Concerning match injury rate, the range was from 2.84 [67] to 47.7 [43] in elite young soccer players, and from 1.8 [92] to 23.5 [70] in non-elite young soccer players.

By contrast, in non-elite young female soccer players, the overall injury incidence ranged from 0.35 [82] to 17.6 [41]. However, it is necessary to emphasise that the overall injury incidence of 0.35 refers only to knee injuries. Only one study [85] reported the overall injury incidence (1.9) in elite young female soccer players, and also in this case the value reported is limited to overuse injuries.

### Severity and re-injury

Twenty-four studies [19, 23, 27, 36, 42–44, 46–48, 50, 53, 56, 59, 65–67, 70–72, 78, 80, 92, 96] reported injury severity in young male soccer players. Nine studies [23, 27, 36, 42–44, 67, 70, 71] recorded the average number of days lost per player. The authors reported a mean between 7 days [43] and 22 days [23]. Six studies [27, 42, 44, 48, 62, 66] classified minimal (slight) the injuries requiring 1–3 days of recovery. The authors reported a range from 7% [48] to 36% [62] of total injuries. Two studies [43, 70], instead, used 0–3 days as limit, and recorded a prevalence of 47% and 70%, respectively.

Nine studies [24, 36, 42–44, 48, 62, 66, 70] reported minor (mild) injuries requiring 4–7 days of recovery and involved 11% [43] to 29% [42] of the players. Five studies [39, 47, 56, 80, 92] included injuries with a range 0–7 days; the percentage reported was from 7% [47] to 43% [92]. Nine studies [27, 43, 44, 47, 48, 56, 62, 66, 70] classified moderate injuries as needing 8–28 days of recovery. The injuries ranged between 16% [43] and 67% [47]. Two studies [36, 42] based on 1–4 weeks classification, reported 43% and 30% of injuries, respectively. Twelve studies [27, 36, 39, 42–44, 47, 48, 56, 62, 66, 70] classified severe (major) injuries as those needing more than 28 days to return to play. The injury rate ranged from 2% [43] to 32% [56]. Three studies [23, 71, 72] calculated the incidence, as reported in Table 3. One study [53] did not specify severity classification.

**TABLE 3:** Injury severity summary in young soccer players. The different lengths of absence employed in the studies were reported.

References	Average n° days lost per player	Minimal Slight	N° days	Minor Mild	N° days	Moderate	N° days	Sever Major	N° Days	Re-injury
**Male**										
Bianco et al. [67]	14			44 [0.53]	1–6 days	52 [0.62]	7–30 days	11 [0.13]	> 30 days	5 (4,67%)
Bowen et al. [23]	22.1	33 [2.9]	1–3 days	33 [2.9]	4–7 days	45 [3.9]	1–4 weeks	27 [2.4]	> 4 weeks	
Read et al. [36]	21.9	118 (14.7%)	2–3 days	164 (20.4%)	4–7 days	345 (42.9%)	1–4 weeks	177 (22%)	> 4 weeks	
Materne et al.[62]		476 (36%)	1–3 days	246 (18.6%)	4–7 days	377 (28.5%)	8–28 days	233 (16.9%)	> 28 days	
Sokka et al. [65]		166 (51%)	0–3 days	61 (19%)	4–7 days	81 (24%)	8–28 days	24 (6%)	> 28 days	
Frisch et al. [70]	21.4	77 (47.3%)	0–3 days	43 (26.4%)	4–7 days	32 (19.6%)	8–28 days	11 (6.7%)	> 28 days	29 (18%)
Le Gall et al. [71]	17.4	153 [1.5]	1–3 days	194 [1.9]	4–7 days	182 [1.7]	1–4 weeks	59 [0.6]	> 4 weeks	18 (3.1%)
Tourny et al. [39]				147 (24,3%)	< 7 days	300 (49.5%)	7–28 days	159 (26.3%)	> 28 days	
Emery et al. [72]		20 [2.85]	< 1 day	10 [1.42]	2–7 days	5 [0.71]	8–14 days	4 [0.57]	> 14 days	
Olumide & Ajide [19] ’		10 (83.3%)	0–3 days	2 (16.7%)	4–7 days		8–28 days		> 28 days	
Le Gall et al. [42]	15	357 (31.0%)	1–3 days	337 (29.3%)	4–7 days	344 (29.9%)	1–4 weeks	114 (9.9%)	> 4 weeks	35 (3%)
Ergün et al. [43]	7.24	31 (70.4%)	0–3 days	5 (11.4%)	4–7 days	7 (15.9%)	8–28 days	1 (2.3%)	> 28 days	11 (25%)
Nogueira et al. [44]	18.6	33 (13.3%)	1–3 days	57 (22.9%)	4–7 days	107 (43.1%)	8–28 days	51 (20.6%)	> 28 days	36 (14.5%)
Brito et al. [46] ^d^		18 (34%)	1–3 days	6 (11%)	4–7 days	21 (40%)	8–28 days	8 (15%)	> 28 days	
Renshaw & Goodwin [47]				9 (7%)	0–7 days	85 (67%)	8–28 days	33 (26%)	> 28 days	
Nilsson et al. [48]		4 (7%)	1–3 days	13 (21%)	4–7 days	25 (41%)	8–28 days	19 (31%)	> 28 days	
Bult et al. [27]	16.8	201 (32.4%)	1–3 days	116 (18.7%)	4–7 days	208 (33.6%)	8–28 days	95 (15.3%)	> 28 days	
Froholdt et al.[78] ^e^		17 (14.8%)	0 days	55 (47.8%)	1–7 days	23 (20%)	8–21 days	20 (17.4%)	> 21 days	
Raya-González et al. [50] ^f^	13	5 (13.2%)	1–3 days	7 (18.4%)	4–7 days	21 (55.3%)	8–28 days	5 (13.2%)	> 28 days	
Raya-González et al. [66]		68 (15%)	1–3 days	85 (18%)	4–7 days	225 (48%)	8–28 days	86 (19%)	> 28 days	
Bacon & Mauger [80]				34 (40.0%)	< 7 days	23 (27.06%)	8–14 days	28 (32.94%)	> 15 days	
Jacobs & Van den Berg [53]		276 (50.7%)	Not reported	137 (25.2%)	Not reported	106 (19.5%)	Not reported	25 (4.6%)	Not reported	
Kakavelakis et al. [56]				62 (30%)	< 7 days	79 (38%)	8–28 days	68 (32%)	> 28 days	
Rössler et al. [59] ^h^	18.9	119 (28.6%)	0–3 days	84 (20.1%)	4–7 days	115 (27.6%)	8–28 days	99 (23.7%)	> 28 days	
Price et al. [96] ^i^		315 (80.6%)	0–6 days	26 (6.6%)	7–13 days	13 (3.3%)	14–29 days	35 (8.9%)	> 30 days	
Nagle et al. [92]				316 (42.5%)	< 1 week	226 (30.4%)	1–3 weeks	49 (6.6%)	> 3 weeks	

**Female**										
Emery et al. [72]		14 [2.02]	< 1 day	14 [2.02]	2–7 days	3 [0.43]	8–14 days	8 [1.15]	> 14 days	
Lislevand et al. [49] ^j^		21 (17%)	0–3 days	2 (2%)	4–7 days	0	8–28 days	0	> 28 days	
Froholdt et al. [78] ^e^		1 (2.6%)	0 days	17 (44.7%)	1–7 days	13 (34.2%)	8–21 days	7 (18.4%)	> 21 days	
O’Kane et al. [88]		91 (52.9%)	1–7 days	29 (16.9%)	8–14 days	25 (14.5%)	15–21 days	27 (15.7%)	> 21 days	
Schiff [57]		4 (9.1%)	1 day	13 (29.6%)	2–4 days	9 (20.5%)	5–10 days	18 (40.8%)	> 10 days	
Nagle et al. [92]				385 (36.7%)	< 1 week	332 (31.7%)	1–3 weeks	65 (6.2%)	> 3 weeks	
Sokka et al.		44 (56%)	0–3 days	20 (26%)	4–7 days	13 (17%)	8–28 days	1 (1%)	> 28 days	

Note: Percentages (%) were reported in parenthesis and incidence [] per 1000h in square brackets preceded by the absolute number.

The re-injury condition was also considered in six studies [42–44, 67, 70, 71], which reported a re-injury rate ranging from 3% [42] to 25% [43].

Seven studies [49, 57, 65, 72, 78, 88, 92] reported injury severity in female soccer players but using different criteria of classification. The results, together with severity recorded during tournaments or for traumatic injuries [78], are presented in Table 3.

### Injury types

The different types of injury were organised as reported in Table 4.

**TABLE 4:** Injury types summary in young soccer players.

References	Age	Maturation	Muscle strain/contracture	Ligament sprain/rupture	Contusion/haematoma/tissue bruising	Fracture/dislocation	Laceration	Growth-related injuries	Overuse	Tendinosis	Joint injury	Other/Unknown
**Male**												

Bianco et al. [67]	U13–U20		93 [1.11]	14 [0.17]								
	U13–U16		63 [1.06]	9 [0.15]								
	U17–U20		30 [1.23]	5 [0.20]								

Bowen et al. [23]	U18–U21		22 [1.9]	35 [3.0]	45 [3.9]	8 [0.7]	2 [0.2]			7 [0.6]	6 [0.5]	8 [0.7]

Deehan et al. [18]			252 (37%)	121 (18%)	71 (10.3%)			23 (3.3%)		41 (5.9%)		

Materne et al. [62]	U9			6 (30%)	7 (35%)	2 (10%)		1 (0.5%)				2 (10%)
	U10			2 (6.9%)	20 (69%)			4 (13.8%)	1 (3.4%)	1 (3.4%)		
	U11			4 (8.3%)	22 (45.8%)	1 (2.1%)		12 (25%)	2 (4.2%)			3 (6.3%)
	U12		1 (2.1%)		23 (48.9%)	2 (4.3%)		12 (25.5%)	2 (4.3%)			
	U13		7 (6.3%)	17 (5.3%)	32 (28.8)	1 (0.9%)		25 (22.5%)	4 (3.6%)	1 (0.9%)		
	U14		12 (6.7%)	14 (7.9%)	38 (21.3%)	10 (5.6%)	2 (1.1%)	44 (24.7%)	11 (6.2%)	1 (0.6%)		3 (1.7%)
	U15		22 (10.3%)	33 (15.4%)	50 (23.4%)	9 (4.2%)	1 (0.5%)	32 (15%)	8 (3.7%)	1 (0.5%)		4 (1.9%)
	U16		22 (8.3%)	42 (15.9%)	77 (29.2%)	1 (0.4%)	1 (0.4%)	35 (13.3%)	16 (6.1%)	1 (0.4%)		2 (0.8%)
	U17		25 (12.3%)	44 (21.6%)	41 (20.1%)	2 (1%)		28 (13.7%)	11 (5.4%)	2 (1%)		5 (2.5%)
	U18		31 (17%)	50 (27.5%)	26 (14.3%)	9 (4.9%)		13 (7.1%)	11 (6.0%)	3 (1.6%)		2 (1.1%)
	U19		6 (24%)	3 (12%)	1 (4%)			2 (8%)	1 (4%)			

Read et al. [36]	U11–U18		162 (20.9%)	136 (17.5%)	57 (7.4%)	25 (3.3%)	18 (2.3%)	51 (6.6%)	33 (4.3%)	33 (4.3%)		209 (27%)

Kemper et al. [69]	U12–U19		18 (13.5%)	22 (16.5%)	29 (21.5%)	3 (2%)		19 (14%)	16 (12%)	6 (4.5%)	9 (7%)	12 (9%)

Frisch et al. [70] ^k^	U15–U19		74 (45.4%)	35 (21.5%)	42 (25.8%)	6 (3.7%)				74 (45.4%)		

Le Gall et al. [71] ^l^		Early maturers	[0.60]					[0.3]		[0.06]		
		Normal maturers	[0.2]					[0.7]		[0.08]		
		Late maturers	[0.08]					[0.9]		[0.02]		

Cezarino et al. [64]	U11–U20		49 (26.2%)	44 (23.5%)	29 (15.5%)	13 (7.1%)				19 (10.2%)		12 (6.4%)

Timpka et al. [12]	U14–U17		2 (5%)	15 (37%)	12 (29%)	6 (15%)	2 (5%)					2 (5%)

Materne et al. [97]		Mature	30 (7.6%)	66 (16.7%)	122 (30.9%)	5 (1.3%)		25 (6.3%)	23 (5.8%)	8 (2%)	1 (0.3%)	8 (2%)
		Early maturers	31 (4.5%)	96 (13.9%)	230 (33.2%)	12 (1.7%)	2 (0.3%)	83 (12%)	43 (6.2%)	6 (0.9%)	1 (0.1%)	21 (3%)
		Normal maturers	28 (6.3%)	44 (9.9%)	156 (35%)	9 (2%)	2 (0.4%)	84 (18.8%)	16 (3.6%)	4 (0.9%)		7 (1.6%)
		Late maturers	2 (6.3%)	2 (6.3%)	11 (34.4%)	1 (3.1%)		6 (18.8%)	2 (6.3%)	1 (3.1%)		1 (3.1%)

Błażkiewicz et al. [38] ^b^	U12–U18		17 (32.7%)	19 (36.5%)		6 (11.5%)	1 (1.9%)				19 (36.6%)	8 (15.4%)

Sullivan et al. [40]	U8–U19		3 (9%)	12 (35%)	13 (38%)	3 (9%)						3 (9%)

Emery et al. [72]	U14–U18		10 [1.42]	10 [1.42]								

Maehlum et al.[41] ^j^	U14–U18			52 (19.5%)	127 (47.7%)	18 (6.8%) 54 (20.3%)						15 (5.6%)

Olumide & Ajide [19]^j^	U11–U19			3 (17.6%)	2 (11.8%)		11 (64.6%)					1 (6.0%)

Le Gall et al. [42]	U14–U16		176 (15.3%)	192 (16.7%)	352 (30.6%)	78 (6.8%)		72 (6.3%)	19 (1.6%)	108 (9.4%)		52 (4.5%)
	U14		53 (12.6%)	76 (18.1%)	109 (26.0%)	27 (6.5%)		50 (11.9%)	5 (1.2%)	55 (13.1%)		14 (3.3%)
	U15		61 (16.9%)	58 (16.1%)	132 (36.6%)	23 (6.4%)		16 (4.4%)	5 (1.4%)	24 (6.6%)		8 (2.2%)
	U16		62 (16.7%)	58 (15.6%	111 (29.9%)	28 (7.6%)		6 (1.6%)	9 (2.4%)	29 (7.8%)		30 (8.1%)

Ergün et al. [43]	U17–U19		27 (61.4%)	4 (9.1%)	9 (20.4%)		1 (2.3%)			1 (2.3%)		
	U17		17 (68%)	4 (16%)	2 (8%)		1 (4%)					
	U18		2 (40%)		3 (60%)							
	U19		8 (57.2%)		4 (28.6%)					1 (7.1%)		

Kuzuhara et al. [21]	U10–U12		1 (3.4%)	5 (17.2%)	8 (27.6%)	5 (17.2%)	5 (17.2%)					5 (17.2%)

Brito et al. [22]	U13–U19		61 (31%)	50 (25%)	45 (23%)	11 (6%)				21 (11%)		11 (6%)
	U13		4 (16%)	4 (16%)	8 (32%)	1 (4%)				8 (32%)		
	U15		14 (34%)	7 (17%)	12 (29%)					3 (7%)		5 (12%)
	U17		17 (30%)	13 (23%)	13 (23%)	6 (10%)				5 (9%)		3 (5%)
	U19		26 (34%)	26 (34%)	12 (16%)	4 (5%)				5 (7%)		3 (4%)

Brito et al. [46]^d^	U13–U19		13 (25%)	8 (15%)	13 (25%)	3 (6%)				7 (13%)		

Renshaw & Goodwin [47]	U9–U18		58 (46%)	20 (16%)						16 (13%)		

Nilsson et al. [48]	U15–U19		31 (53%)	15 (24%)								

Bult et al.[27]	U12–U19		173 (27.9%)	78 (12.6%)	174 (28.1%)	56 (9.1%)				81 (13.1%)		

Froholdt et al. [78]^e^	U6–U16		23 (20%)	24 (20.9%)	50 (43.5%)	6 (5.2%)						12 (10.4%)
	U6–U12		7 (16%)	10 (23%)	17 (39%)	3 (7%)						7 (16%)
	U13–U16		16 (23%)	14 (20%)	33 (46%)	3 (4%)						5 (7%)

Khodaee et al. [51]	U14–U18		504 (17.3%)	697 (23.9%)	421 (14.4%)	262 (9.0%)						534 (18.3%)

Herdy et al. [52]	U11–U20		64 (32%)	54 (27%)	62 (31%)					10 (5%)		
	U11		3 (25%)	2 (16%)	7 (59%)							
	U13		6 (39%)	3 (18%)	4 (27%)					1 (6%)		
	U15		17 (37%)	9 (19%)	15 (32%)					2 (4%)		
	U17		24 (37%)	18 (28%)	19 (29%)					3 (4%)		
	U20		14 (22%)	23 (38%)	17 (28%)					4 (7%)		

Hoff & Martin, [79]	U8–U16		28 (23.3%)	46 (38.3%)	22 (18.3%)	8 (6.7%)						16 (13.4%)

Bacon & Mauger, [80]	U18–U21		12 (14.12%)	16 (18.82%)	17 (20%)	5 (5.88%)			16 (18.82%)			7 (8.24%)

McCarroll et al. [54]	U10–U19		17 (9.7%)	47 (26.7%)	44 (25.0%)	22 (12.5%)						41 (23.3%)

Andreasen et al. [55] ^m^	U10–U19		26 (27.1%)	26 (27.1%)	38 (39.6%)	20 (20.8%)						12 (12.5%)

Kakavelakis et al. [56]	U12–U15		49 (23%)	69 (33%)	43 (21%)	16 (8%)				15 (7%)	6 (3%)	7 (3%)

Rössler et al. [59] ^h^	U8–U12		70 (16.8%)	86 (20.6%)	94 (22.5%)	44 (13%)	9 (2.1%)	2 (0.5%)	27 (6.5%)	7 (1.7%)		38 (9.1%)
	U8		7 (12.5%)	14 (25.0%)	12 (21.4%)	9 (16.1%)	3 (5.4%)		3 (5.4%)	1 (1.8%)		6 (10.7%)
	U10		11 (9.6%)	25 (21.7%)	27 (23.5%)	15 (13.0%)	2 (1.8%)	2 (1.7%)	9 (7.8%)	2 (1.7%)		13 (11.3%)
	U12		52 (21.1%)	47 (19.1%)	55 (22.4%)	30 (12.2%)	4 (1.6%)		15 (6.1%)	4 (1.6%)		19 (7.7%)

Volpi et al. [60]	U10–U19		7 (9.7%)	23 (31.9%)		16 (22.2%)	2	:2 (30.6%)		4 (5.6%)		

**Female**												

Emery et al. [72]	U14–U18		9 [1.29]	17 [2.44]								

Maehlum et al[41]^j^	U14–U18			37 (25.5%)	66 (45.5%)	9 (6.2%)						13 (9.0%)

Del Coso et al. [45]	U18		79 (8.7%)	347 (38.4%)	233 (25.8%)	78 (8.6%)	5 (0.5%)			45 (5%)		

Lislevand et al. [49] ^j^	U13-O16		1 (0.8%)	14 (11.7%)	68 (56.7%)		27 (22.5%)		3 (2.5%)			7 (5.8%)

Froholdt et al.[78] ^e^	U6–U16		3 (7.9%)	15 (39.5%)	13 (34.2%)	2 (5.3%)						5 (13.1%)
	U6–U12		1 (9%)	3 (27%)	5 (45%)							2 (18%)
	U13–U16		2 (7%)	12 (44%)	8 (30%)	2 (7%)						3 (11%)

Khodaee et al.[51]	U14–U18		488 (15%)	1115 (34.4%)	337 (14.4%)	194 (6.0%)						493 (15.2%)

Watson et al. [81]	U13–U18		3 (11%)	18 (65%)	2 (7%)							

Steffen et al. [86]	U14–U16		77 (23.3%)	141 (42.7%)	78 (23.6%)	9 (2.7%)						24 (7.3%)

O’Kane et al. [88]	U11–U15		[08 (62.4%) +	108 (62.4%) +	52 (30%)	7 (4%)						5 (2.9%)

Andreasen et al.[55]^j^	U10–U19		14 (39.9%)	14 (38.9%)	11 (30.6%)	5 (13.9%)						6 (16.7%)

Watson et al. [90]	U16		6 (17%)	22 (61%)	3 (8%)	1 (3%)						

Note: Percentages (%) were reported in parenthesis and incidence [] per 1000h in square brackets preceded by the absolute number. + The authors grouped sprain and strain together.

In male youth soccer players, muscle strains/contractures were recorded, with a percentage ranging between 3% [21] and 61% [43]. Ligament sprains/ruptures involved a number of players ranging from 9% [43] to 38% [79]. Contusions, combined with haematoma and tissues bruising, recorded a percentage prevalence between 7% [36] and 38% [40]. Fractures and dislocations affected young soccer players with a percentage between 2% [69] and 22% [60]. Six studies [12, 21, 36, 38, 43, 59] recorded laceration, as well, with a prevalence from 2% [38] to 17% [21]. Growth related injuries fluctuated from 3% [18] to 31% [60], and tendinosis from 2% [59] to 13% [27]. Only three studies [38, 56, 69] recorded joint injuries.

In young female soccer players, muscle strains and contractures recorded injury prevalence between 9% [45] and 23% [86]. Five studies [45, 51, 81, 86, 90] presented ligament sprains/ruptures; the percentage ranged from 34% [51] to 65% [81]. Contusions combined with haematomas and tissue bruising were in a range between 7% [81] and 26% [45]. Five authors [45, 51, 86, 88, 90] included fractures and dislocations; the value ranged between 3% [86] and 9% [45].

Studies reporting injury incidence [23, 67, 72], and data recorded according to chronological age [22, 42, 43, 52, 59, 67, 78], biological age [71, 97] or specific cases such as data collected during tournament [19, 36, 41, 49] are presented in Table 4.

### Injury mechanisms

Considering the injury mechanism, fifteen studies [12, 18, 21, 23, 39, 43, 44, 47, 51, 56, 60, 70, 72, 80, 94] reported comparison between contact and non-contact injuries in male youth soccer players. The percentage ranged from 23% [39] to 72% [21] for contact injuries and from 7% [21] to 77% [39] for non-contact injuries. Three studies [27, 60, 69] made a distinction between traumatic and overuse injury, reporting a percentage ranging from 65% [60] to 76% [27] for traumatic injuries and from 25% [27] to 35% [60] for overuse injuries. Four studies [44, 51, 56, 70] showed only overuse/progressive injuries and the percentage ranged from 5% [70] to 20% [56]. Four studies [24, 45, 51, 88] made a comparison between contact and non-contact injuries in female youth soccer players. The range was from 22% [45] to 69% [51] for contact injuries and from 22% [51] to 78% [45] for non-contact injuries. Two authors [24, 86] investigated traumatic and overuse injuries, as well. The percentage values were 78% and 87%, respectively, for traumatic injuries, and 22% and 13%, respectively, for overuse injuries.

Injury mechanism data are presented in Table 5 according to chronological age, biological age, and for specific cases such as tournament injuries [19, 49] or specific anatomical areas [37, 87].

**TABLE 5:** Injury mechanisms summary in young soccer players.

References	Age	Biological age	Contact injuries	Non-contact injuries	Traumatic/acute	Progressive injury/Overuse	Unkown
**Male**							

Bowen et al. [23]	U18—U21		59 [5.2]	79 [6.9]			

Deehan et al. [18]	U9—U19		210 (31%)	475 (69%)			

Cloke et al. [37]^a^	U9–U18		24 (42.1%)	32 (57.9%)			

Kemper et al. [69]	U12—U19				88 (65.7%)	46 (34.3%)	

Frisch et al. [70]	U15—U19		60 (36.8%)	95 (58.3%)		8 (4.9%)	

Timpka et al. [12]			30 (68%)	14 (32%)			

Tourny et al. [39]	U12–U15		34 (23%)	114 (77.0%)			
	U16–U20		154 (34.4%)	294 (65.6%)			

Emery et al. [72]	U14—U18		36 (46.15%)	42 (53.85%)			

Olumide & Ajide [19] ’	U11—U19		14 (93.3%)	1 (6.7%)			
Ergün et al. [43]	U17—U19		14 (60.9%)	9 (39.1%)			
	U17		5 (38.5%)	8 (61.5%)			
	
	U18		3 (100%)				
	U19		6 (85.7%)	1 (14.3%)			

Kuzuhara et al. [21]	U10—U12		21 (72.4%)	2 (6.9%)			6 (20.7%)

Nogueira et al. [44]	U17—U19		81 (32.8%)	109 (44%)		32 (12.8%)	26 (10.4%)
	U17		47 (19%)	57 (23%)		21 (8.4%)	13 (5.2%)
	U19		34 (13.8%)	52 (21%)		11 (4.4%)	13 (5.2%)

Brito et al. [22]	U13				[0.4]	[0.3]	
	U15				[0.6]	[0.6]	
	
	U17				[1.0]	[0.7]	
	U19				[1.1]	[0.8]	

Sieland et al. [94]	U12—U19		72 (58%)	53 (42%)			

Renshaw & Goodwin [47]	U9–U18		36 (28%)	91 (72%)			

Materne et al. [97]		Mature	146 (37%)	249 (63%)			
		Early maturers	287 (41.5%)	405 (58.5%)			
	
		Normal maturers	187 (41.9%)	259 (58.1%)			
		Late maturers	12 (37.5%)	20 (62.5%)			

Bult et al. [27]	U12—U19				468 (75.5%)	152 (24.5%)	

Froholdt et al. [78] e	U6—U16		72 (62.6%)	43 (37.4%)			
	U6–U12		28 (64%)	16 (36%)			
	U13–U16		44 (62%)	27 (38%)			

Khodaee et al. [51]	U14—U18		1971 (67.7%)	679 (23.3%)		210 (7.2%)	52 (1.8%)

Bacon & Mauger [80]	U18—U21		36 (42.35%)	44 (51.76%)			5 (5.88%)

Kakavelakis et al. [56]	U12—U15		132 (63.4%)	36 (17%)		41 (19.6%)	

Rössler et al. [59] h	U8–U12		239 (57.3%)	87 (20.9%)		50 (12.0%)	20 (4.8%)
	U8		33 (59%)	9 (16%)		8 (14.3%)	5 (8.9%)
	
	U10		74 (64.3%)	19 (16.5%)		16 (13.9%)	4 (3.5%)
	U12		132 (53.7%)	59 (23.9%)		26 (10.6%)	11 (4.5%)

Volpi et al. [60]	U10—U19		26 (36.2%)	46 (63.8%)	47 (65.2%)	25 (34.8%)	

**Female**							

Soligard et al. [24]	U16		133 (51%)	115 (44%)	203 (78%)	56 (22%)	11 (5%)

Del Coso et al. [45]	U18		195 (21.6%)	709 (78.4%)			

Schiff et al. [58]	U12—U14				27 (4.7)	17 (2.9)	

Lislevand et al. [49] ^j^	U13—O16		106 (94%)	7 (6%)			
	U13		43 (96%)	2 (4%)			
	U16		40 (93%)	3 (7%)			
O16		23 (92%)	2 (8%)			

Froholdt et al. [78] ^e^	U6–U16		23 (60.5%)	15 (39.5%)			
	U6–U12		7 (64%)	4 (36%)			
	U13—U16		16 (59%)	11 (41%)			

Khodaee et al. [51]	U14—U18		2249 (69.4%)	700 (21.6%)		226 (7.0%)	67 (2.1%)

Steffen et al. [86]	U14—U16				330 (86.8%)	50 (13.2%)	

Clausen et al. [87] ^g^	U15—U18		16 (47%)	7 (20.5%)	23 (67.6%)	11 (32.3%)	

O’Kane et al. [88]	U11—U15		115 (66.5%)	58 (33.5%)			

Note: Percentages (%) were reported in parenthesis and incidence [] per 1000h in square brackets preceded by the absolute number.

### Anatomical location

The anatomical districts of injuries were organised as reported in Table 6. When the articles presented data for individual anatomical areas, they were grouped by the authors reporting the overall percentage. Head, neck, and cervical spine injuries were not reported.

**TABLE 6:** Anatomic location summary of injuries in young soccer players

References	Age	Lower extremities	Ankle and foot	Lower leg/Calf/Achilles tendon	Knee	Posterior thigh	Anterior thigh	Thigh	Groin/adductors/pelvis/hips	Upper body/ Abdomen/ lower back/trunk	Arm/shoulder/hand/wrist	Other
**Male**												

Elias [35]^j^	U12–U19		598 (25.4%)	216 (9.2%)	348 (14.8%)			304 (12.9%)		194 (8.2%)	60 (2.5%)	
	U12		72 (24.2%)	25 (8.4%)	57 (19.1%)			32 (10.7%)		22 (7.4%)	5 (1.7%)	
	U14		155 (25.6%)	58 (9.6%)	76 (12.5%)			77 (12.7%)		66 (10.9%)	9 (1.5%)	
	U16		190 (24.1%)	70 (8.9%)	118 (15.0%)			117 (14.8%)		65 (8.2%)	26 (3.3%)	

Wik et al. [61]	U13–U18		247 (22%)	100 (9%)	145 (13%)			274 (25%)	159 (14%)	62 (6%)	97 (9%)	
	U19		181 (29.0%)	63 (10.1%)	97 (15.5%)			78 (12.5%)		41 (6.6%)	20 (3.2%)	

Bianco et al. [67]	U13–U20			12 [0.14]	19 [0.22]			36 [0.43]	23 [0.27]			
	U13–U16			7 [0.12]	12 [0.20]			23 [0.38]	18 [0.30]			
	U17–U20			5 [0.20]	7 [0.28]			13 [0.53]	5 [0.20]			

Bowen et al. [23]	U18–U21		54 [4.7]	4 [0.4]	19 [1.7]	10 [0.9]	11 [1.0]		18 [1.6]	4 [0.4]	8 [0.7]	

Deehan et al. [18]	U9–U19		165 (24%)		102 (15%)			211 (31%)	44 (6.5%)	69 (10%)		94 (13.5%)

Cezarino et al. [64]	U11–U20		42 (22.5%)	8 (4.2%)	43 (23%)			48 (25.7%)	22 (11.8%)	7 (3.7%)	2 (1%)	

Read et al. [36]	U11–U18		206 (25.7%)	17 (2.1%)	161 (20.0%)	49 (6.1%)	76 (9.5%)		113 (14.1%)	48 (6.0%)	51 (6.3%)	11 (1.4%)

Frisch et al. [70]	U15–U19		38 (23.3%)	5 (3.1%)	28 (17.2%)			63 (38.7%)	8 (4.3%)	9 (5.5%)	7 (4.2%)	

Timpka et al. [12]	U14–U17	25 (58%)	13 (32%)	2 (5%)	4 (10%)	1 (2%)	2 (5%)		5 (7%)	4 (10%)	5 (12%)	

Błażkiewicz et al. [38] ^b^	U12–U18	29 (55.8%)								9 (17.3%)	18 (34.6%)	

Tourny et al. [39]	U12–U15	130 (87.7%)	17 (10.4%)		24 (14.7%)			38 (23.3%)	31 (19.0%)	12 (7.4%)	4 (2.5%)	
	U16–U20	415 (92.6%)	81 (26.6%)	27 (6.0%)	53 (11.8%)			144 (32.1%)	72 (16.1%)	12 (2.7%)	18 (4.0%)	

Emery et al. [72]	U14–U18		11 (1.56)	5 (0.71)	4 (0.57)			2 (0.28)	3 (0.43)	2 (0.28)		

Maehlum et al. [41]^j^	U14–U18	159 (59.8%)								16 (6.0%)	37 (13.9%)	

De Ridder et al. [74]	U11–U17		12 (18%)	6 (9%)		4 (6%)	12 (18%)		13 (19%)			

Olumide & Ajide [19] ’	U11–U19		1 (6.0%)		3 (17.6%)			3 (17.6%)	3 (17.6%)		6 (35.2%)	

Le Gall et al. [42]	U14–U16		300 (26%)	60 (5.2%)	176 (15.3%)			282 (24.5%)	82 (7.1%)	113 (9.8%)	119 (10.3%)	
	U14		116 (27.6%)	24 (5.7%)	74 (17.6%)			89 (21.2%)	33 (7.9%)	31 (7.4%)	48 (11.5%)	
	U15		82 (22.7%)	19 (5.3%)	49 (13.6%)			107 (29.6%)	24 (6.7%)	36 (10.0%)	35 (9.7%)	
	U16		102 (27.5%)	17 (4.6%)	53 (14.3%)			86 (23.2%)	25 (6.8%)	46 (12.4%)	36 (9.8%)	

Materne et al. [62]	U9		10 (50%)	3 (15%)	4 (20%)						3 (15%)	
	U10		9 (31%)	4 (13.7%)	9 (31%)			4 (13.7%)			3 (10.3%)	
	U11		22 (45.8%)	5 (10.5%)	9 (18.8%)			5 (10.5%)	3 (6.3%)	1 (2.1%)	3 (6.3%)	
	U12		10 (21.3%)	10 (21.2%)	6 (12.8%)			9 (19.1%)	4 (8.5%)	3 (6.4%)	5 (10.6%)	
	U13		25 (22.5%)	20 (18%)	16 (14.4%)			23 (19.8%)	7 (6.3%)	6 (5.4%)	13 (11.7%)	
	U14		32 (19.6%)	30 (16.8%)	15 (8.4%)			46 (25.8%)	29 (16.3%)	9 (5.1%)	8 (4.5%)	
	U15		52 (24.3%)	25 (11.6%%)	16 (7.5%)			51 (23.8%)	32 (15%)	18 (8.4%)	9 (4.2%)	
	U16		70 (26.5%)	31 (11.8%)	34 (12.9%)			64 (23.1%)	37 (14%)	17 (6.5%)	5 (1.9%)	
	U17		53 (26%)	11 (5.4%)	26 (12.7%)			59 (27.5%)	37 (18.1%)	6 (3%)	6 (3%)	
	U18		43 (26.6%)	12 (6.6%)	31 (17%)			59 (32.4%)	18 (9.9%)	7 (3.8%)	8 (4.4%)	
	U19		5 (20%)	4 (16.0%)	31 (17.0%)			9 (13.3%)	4 (16%)	1 (4%)		

Schmidt-Olsen et al. [20]	U13–U17		73 (23.4%)	34 (10.9%)	81 (26%)				28 (8.9%)	43 (13.8%)	32 (10.3%)	
	U13		30 (21.8%)	12 (8.8%)	39 (28.5%)				15 (11%)	18 (13.1%)	12 (8.8%)	
	U15		13 (19.4%)	10 (15.0%)	17 (25.3%)				3 (4.5%)	13 (19.4%)	8 (11.9%)	
	U17		30 (27.7%)	12 (11.1%)	25 (23.1%)				12 (11.1%)	12 (11.1%)	11 (11%)	

Ergün et al. [43]	U17–U19		5 (11.4%)	2 (4.6%)	3 (6.8%)			14 (31.8%)	11 (25%)	6 (13.6%)		
	U17		2 (8%)	2 (8%)	2 (8%)			8 (32%)	8 (32%)	1 (4%)		
	U18		2 (40%)							3 (60%)		
	U19		1 (7.1%)		1 (7.1%)			6 (42.9%)	3 (21.5%)	2 (14.3%)		

Kuzuhara et al. [21]	U10–U12	15 (51.7%)								2 (6.9%)	4 (13.8%)	3 (10.3%)

Nogueira et al. [44]	U17–U19		64 (25.8%)	18 (7.3%)	34 (13.7%)	31 (12.5%)	30 (12.1%)	61 (24.6%)	35 (14.1%)	22 (8.9%)	13 (5.2%)	

Brito et al. [22]	U13–U19	172 (86%)	61 (31%)	14 (7%)	24 (12%)			60 (30%)	14 (7%)	8 (5%)	14 (7%)	
	U13		10 (39%)	2 (8%)	3 (13%)			6 (25%)	2 (8%)	1 (4%)	1 (4%)	
	U15		7 (17%)	3 (7%)	4 (10%)			14 (34%)	4 (10%)	6 (15%)	2 (5%)	
	U17		13 (23%)	7 (12%)	9 (16%)			15 (26%)	5 (9%)	1 (2%)	6 (11%)	
	U19		31 (40%)	2 (3%)	8 (11%)			25 (33%)	3 (4%)		5 (6%)	
Brito et al. [46] ^d^	U13–U19		16 (30%)	5 (9%)	5 (9%)			12 (23%)	4 (8%)	6 (11%)	3 (6%)	

Renshaw & Goodwin [47]	U9–U18		22 (17.3%)	7 (5.5%)	22 (17.3%)	17 (13.4%)	27 (21.6%)		17 (13.4%)	6 (4.7%)	6 (4.7%)	

Nilsson et al. [48]	U15–U19		12 (19.7%)	4 (6.6%)	5 (8.2%)			16 (26.2%)	20 (32.8%)	1 (1.6%)	3 (4.9%)	

Bult et al. [27]	U12–U19											

Froholdt et al. [78] ^e^	U6–U16		35 (30.4%)	13 (11.3%)	12 (10.4%)			18 (15.63%)	12 (10.4%)	9 (7.8%)		
	U6–U12		17 (38%)	5 (11%)	7 (16%)			4 (9%)	2 (5%)	2 (5%)		
	U13–U16		18 (25%)	8 (11%)	5 (7%)			14 (20%)	10 (14%)	7 (10%)		

Khodaee et al. [51]	U14–U18		723 (24.8%)	229 (7.9%)	409 (14.1%)			363 (12.5%)	148 (5.1%)	140 (4.8%)	286 (9.8%)	24 (0.8%)

Hoff & Martin [79]	U8–U16	72 (60%)								15 (12.5%)	17 (14%)	

Bacon & Mauger [80]	U18–U21		32 (37.65%)	2 (2.35%)	14 (16.47%)	3 (3.53%)	6 (7.06%)		15 (17.65%)	6 (7.06%)	2 (2.35%)	2 (2.35%)

Jacobs & Van den Berg [53]			145 (26.6%)	12 (2.2%)	52 (9.5%)				77 (14.1%)	9 (1.6%)	128 (23.5%)	

McCarroll et al. [54]	U10–U19		44 (25%)	44 (25%)	25 (14.2%)	2 (1.1%)	21 (11.9%)		5 (2.8%)	2 (1.1%)	13 (7.4%)	

Andreasen et al. [55] ^j^	U10–U19	71 (73.9%)								3 (3.1%)	13 (13.5%)	

Kakavelakis et al. [56]	U12–U15		60 (29%)	13 (6%)	75 (36%)			19 (9%)		11 (5%)	25 (12%)	

Rössler et al. [59]^h^	U8–U12		139 (33.4%)	29 (7.0%)	68 (16.3%)			41 (9.8%)	41 (9.8%)	8 (2%)	65 (15.6%)	
	U8		26 (46.4%)	2 (3.6%)	6 (10.7%)			4 (7.1%)	5 (8.9%)		8 (14.3%)	
	U10		44 (38.2%)	10 (8.7%)	19 (16.5%)			7 (6.1%)	8 (7.0%)	2 (1.8%)	14 (12.2%)	
	U12		69 (28.1%)	17 (6.9%)	43 (17.5%)			30 (12.2%)	28 (11.4%)	6 (2.4%)	43 (17.5%)	

Price et al. [96]^i^	U5–U18		15 (3.8%)	59 (15.1%)	75 (19.1%)			17 (4.3%)	77 (19.6%)	42 (10.7%)	76 (19.4%)	

Volpi et al. [60]	U10–U19		15 (20.8%)	2 (2.8%)	30 (41.7%)			7 (9.7%)	8 (11.1%)		9 (12.5%)	

Nagle et al. [92]	U14–U18		(35.4%)		(26.3%)			(23.1%)				

**Female**												

Elias [35]^j^	U12–U19		394 (28.4)	104 (7.5%)	271 (19.5%)			106 (7.6%)		196 (14.1%)	24 (1.7%)	
	U12		46 (24.1%)	12 (6.3%)	43 (22.5%)			12 (6.3%)		14 (7.3%)	4 (2.1%)	
	U14		113 (25.7%)	27 (6.1%)	82 (18.7%)			33 (7.5%)		41 (9.3%)	11 (2.5%)	
	U16		162 (31.7%)	37 (7.2%)	96 (18.8%)			38 (7.4%)		35 (6.8%)	6 (1.2%)	
	U19		73 (29.7%)	28 (11.4%)	50 (20.3%)			23 (9.3%)		16 (6.5%)	3 (1.2%)	

Emery et al. [72]	U14–U18		17 [2.44]	1 [0.14]	11 [1.58]			3 [0.43]	5 [0.72]	1 [0.14]	3 [0.43]	

Maehlum et al. [41]^j^	U14–U18			159 (59.8%)						15 (10.3%)	21 (14.4%)	

Del Coso et al. [45]	U18		241 (26.6%)	77 (8.5%)	270 (29.9%)			73 (8.1%)	18 (2%)	45 (5%)	131 (14.5%)	

Lislevand et al. [49]^j^	U13–O16		44 (36.7%)	9 (7.5%)	30 (25%)			9 (7.5%)	6 (5%)	3 (2.5%)	18 (15%)	

Froholdt et al. [78]^e^	U6–U16		13 (34.2%)	1 (2.6%)	8 (6.9%)			3 (7.9%)	1 (2.6%)	3 (7.9%)		
	U6–U12		2 (18%)	1 (9%)	3 (27%)				1 (9%)	1 (9%)		
	U13–U16		11 (41%)		5 (19%)			3 (11%)		2 (7%)		

Khodaee et al. [51]	U14–U18		880 (27.2%)	222 (6.9%)	637 (19.7%)			339 (10.5%)	93 (2.9%)	97 (3.0%)	218 (6.8%)	19 (0.6%)

Watson et al. [81]	U13–U18		13 (47%)		5 (18%)			3 (11%)		2 (7%)		

O’Kane et al. [82] ^i^	U12–U15		16 (19.8%)	7 (8.6%)	38 (46.9%)			4 (4.9%)	16 (19.8%)			

Steffen et al. [86]	U14–U16		147 (44.6%)	17 (5.2%)	53 (16.1%)			49 (14.8%)	23 (7%)	27 (8.2%)		

O’Kane et al. [88]	U11–U15		84 (48.5%)	9 (5.2%)	43 (24.9%)			19 (11%)	18 (10.4%)			

Andreasen et al. [55]^j^	U10–U19	23 (63.9%)								4 (11.1%)	6 (16.7%)	

Schiff [57]	U11–U14	35 (77.5%)								4 (8.9%)	6 (13.3%)	

Watson et al. [90]	U16		17 (47%)	1 (3%)	7 (19%)					2 (6%)	4 (11%)	

Nagle et al. [92]	U14–U18		(37.7%)		(33.4%)			(17.2%)				

Note: Percentages (%) were reported in parenthesis and incidence [] per 1000h in square brackets preceded by the absolute number ^a^ The authors report only ankle injuries.

^a^ The authors report only ankle injuries; ^b^ The authors report only goalkeeper injuries; ^c^ Data refer only to non-contact injuries; ^d^ The authors report only preseason injuries; ^e^ Data refer to traumatic injuries; ^f^ The authors report only muscle injuries; ^g^ Data refer to knee injuries; ^h^ Data refer to a mixed sample, male and female; ^i^ The authors report only overuse injuries; ^j^ Data were recorded during tournament; ^k^ The authors combine muscle and tendon injuries; ^l^ Data refer to groin strain; ^m^ The authors combine sprain and strain.

In general, in male youth soccer players, five studies [21–23, 41, 79] reported an overall prevalence of lower extremity injuries. They were the most common anatomical injured district with a percentage ranging between 52% [21] and 93% [39]. In particular, ankle and foot in conjunction registered a prevalence ranging between 10% [39] to 38% [80]. Several studies recorded lower leg (calf/ Achilles tendon) injuries. The percentage ranged between 2% [36] and 25% [54]. The knee registered a range between 7% [43] and 42% [60]. Twelve studies [18, 39, 42–44, 48, 51, 56, 60, 67, 70, 92] reported the overall percentage of injuries in the thigh. The range was from 9% [56] to 39% [70]. Specifically, the prevalence ranged from 5% [12] to 22% [47] for the anterior thigh and from 1% [54] to 13% [47] for the posterior thigh.

Hip injuries, including groin, adductors, and pelvis injuries, recorded a percentage ranging between 3% [54] and 33% [48]. For the upper body, the range was from 1% [54] to 14% [20]. The upper extremities, including the arm, shoulder, wrist, hand, and fingers, recorded a range from a minimum of 2% [80] to a maximum of 23% [53].

In women’s youth soccer, the ankle and foot were affected with a percentage ranging between 27% [45] and 49% [88]. The injury incidence recorded was 2.44 [72]. Six studies [45, 51, 57, 81, 86, 88] reported injury prevalence in the lower leg with a percentage ranging from 3% [90] to 9% [45]. For the knee, the value ranged from 16% [86] to 33% [92]. Six studies [45, 51, 81, 86, 88, 92] identified thigh injuries with a range from 8% [45] to 17%. Four studies [45, 51, 86, 88] recorded injuries in the hip area with a percentage from 2% [45] to 20% [88] and an injury incidence of 0.72 [72]. For the upper body, the range was from 3% [51] to 9% [57], while for upper extremities it was from 7% [51] to 15% [45].

Injury prevalence according to chronological age and for specific cases such as goalkeeper injuries [38], only traumatic injuries [78], and preseason injuries [46], is presented in Table 6.

## DISCUSSION

Although soccer is generally considered a safe sport, much attention has been placed during the last years on injury risk in young soccer players. The need to reduce medical health care costs and to promote talent development prompted investigation of the injury rate and risk factors related to it. Following the suggestion of Van Mechelen et al. [17], before introducing adequate prevention strategies, it is necessary to describe sports injuries and to identify the mechanism underlying them.

**FIG. 2:**
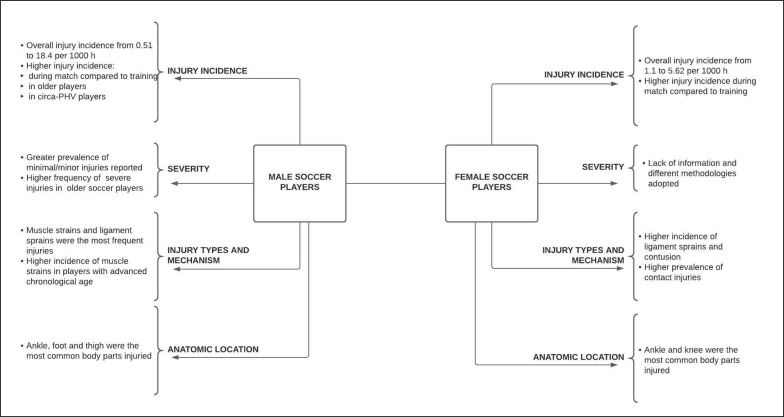
Summary of the main results.

Therefore, the aim of this systematic literature review was to summarise and to show a broad view of injury incidence and injury risk factors in young soccer players regardless of different injury definitions or different sample characteristics such as chronological age, biological age, sex, or level of play. According to the authors’ best knowledge, this is the first review that tries to build a general overview of the risk of injury in young soccer players combining epidemiological data and injury risk factors together. The main results are summarised in Figure 2.

### Injury definition and collection process

The absence of standardization in the research method produces confusion and difficulty in the interpretation of the results [99, 101]. In order to avoid this bias, Fuller et al. [98], introduced the consensus statement on injury definitions and data collection procedures. However, even after the publication of the consensus statement, many studies continued to use different definitions [21, 50, 74, 77]. This helps to explain the high variability of the results found in the current review. In general, the two main strategies adopted to detect injury condition were time loss [18, 36, 42, 61, 73] and medical attention required [7, 12, 20, 72]. The latter method allows attention to be paid to a higher number of injuries, with the risk of overestimating or including injuries not clinically relevant. The time-loss method, on the other hand, is mainly based on days of absence, but also in this case, the use of different time windows, from 24 h [51, 56] up to four weeks [96], increases the variability of results.

The collection process strategies were closely connected with the experimental design. Even in this case, the adoption of different data collection methods could produce discrepancy in results [102, 103]. Physiotherapists, physicians and medical staff have been widely recruited for prospective cohort studies [18, 23, 48, 68, 75]; however, few studies [51, 53, 56, 57, 79, 83, 85, 90] adopted questionnaires or web data collection tools. Schiff et al. [58] found high agreement between certified athletic trainers and an Internet-based survey about the injury collection process. Support from parents in reporting injuries could be a valid alternative to the medical staff, especially in clubs with limited economic resources; in spite of this, the possibility of subjective answers and the inability to discriminate different kinds of injuries remain to be considered.

In retrospective studies [38, 53, 57, 79], self-administered questionnaires were mainly used. This method allows one to collect injury data quickly without requiring all the time necessary for prospective studies. In this case, there are some limits connected with recall bias [103, 104] and inability to report incidence or severity [105]. Therefore, authors should be aware that the choice of injury definition and data collection method could impact the quality of the epidemiological analysis [103].

### Injury incidence and severity

The overall injury incidence per 1000 h ranged from 0.51 [40] to 18.4 [43] in male youth soccer players, with an average number of injuries between 0.6 [18] and 2.5 [71] per player. The high variability, as previously mentioned, could be explained by the different injury definitions, collection processes, and sample characteristics. Although the absolute number of injuries recorded during matches and training was similar [18, 21] or sometimes higher during training [48, 53], the injury incidence was considerably higher during matches [21, 23, 42, 69]. The competition subjects players to greater physiological and psychological demands, unlike training, during which the aim is to improve performance and to reduce the risk of injury [39]. This is better shown during youth soccer tournaments, where the authors found injury incidence up to 113.6 per 1000 h [19]. Tournaments are generally played in few days, and every match is crucial to reach the final stage. The high density of matches, with a short recovery time in between, and the technical and tactical demands, further contribute to increase the risk of injury. As evidence of this, Maehlum et al. [41] reported an injury rate that increased during the final rounds, played in knockout, compared to opening rounds.

Another factor that explains the variability of the results is the different players’ chronological age considered in the studies. Soccer players aged from 5 to 18 years old were included in the analysis. It is well known that this age group encompasses different development stages passing from childhood to adolescence. In this period of their life, athletes experience rapid changes – psychological, physiological, cognitive, and behavioural – accompanied by an increase in weight, height, muscle mass and changes in body composition [106]. This anthropometric growth, together with hormonal and motor control changes, may produce different injury predispositions in young soccer players [73]. Many studies [20, 36, 42, 61, 76, 78] reported an increase in injury rate with chronological age. As the years passed, players become faster and stronger [78] and they are subjected to higher volume of exposures and intensity of competition than in the past, and all these factors may contribute to increased injury incidence. However, if it is true that the overall injury incidence increases with age, it seems that training injury incidence was higher in younger soccer players [42, 43, 64], who, lacking technical and tactical skills, may be more susceptible to a higher risk of injury [42, 43]. However, not all the studies reported significative differences according to chronological age [44, 67].

As mentioned before, adolescence is a period characterised by rapid psychological and physiological changes; therefore, players with the same chronological age could experience different stages of puberty. For this reason, a few studies tried to investigate how biological age impacts on the injury rate. In this regard, three studies [71, 77, 97] adopted X-ray assessment to identify early, normal, and late maturer players. Materne et al. [97] found the greatest overall injury risk in early maturer players. Le Gall et al. [71] did not find a significant difference in overall injury incidence, but observed that early and late maturers may be vulnerable to different types of injuries. Johnson et al. [77] found more injuries in early maturers than in late or normal maturers, but the analysis did not reveal differences after adjusting for training time, playing time, height, and position played. Further analysis is needed to clarify whether early or late maturation may impact on injury predisposition. However, greater agreement was found in studies [27, 73, 89] adopting the maturity offset method to assess maturity timing [107]. Particularly, a higher injury incidence was found in the period labelled as circa-PHV, which is the period characterised by a rapid growth spurt. In this particular period known as “adolescent awkwardness”, the motor control strategies are altered [108], and the rapid growth of anatomical structures such as tendons, ligaments and bones may predispose soccer players to a higher risk of injury. Only one study adopted the Khamis Roche equation to assess the status of maturation of the players [63]. The authors observed increased injuries in players classified as “early-maturing” compared to “on-time” or “late-maturing” players.

Chronological and biological ages also appear to affect the severity of injuries. The different time intervals used to classify minimal, minor, moderate, and severe injuries make the comparison between the reported studies difficult. However, most studies found a greater prevalence of minimal/minor injuries [39, 42, 43, 51, 53, 56, 62, 70, 71, 78] with a mean recovery time requiring less than one week. A few studies [12, 44] recorded a high prevalence of severe injuries. Moreover, severe injuries may be more frequent in older players [22, 39, 67]. This result is in line with previous studies conducted on adult soccer players [109, 110]. The re-injury rate reported was low, almost always close to 3% [22, 42, 44, 67, 71], and in a few cases higher than 15% [43, 70]. The re-injury rate may be affected by the presence or absence of a team’s medical staff, and by imposed pressures to return to play, particularly in elite and older categories. Furthermore, only four studies [27, 61, 62, 66] reported injury burden, which is the result of combination of severity and incidence. The highest injury burden was found in U16 [27, 61, 66] and U18 soccer players [62]. This may be explained by the rapid changes in height and weight that characterise these age groups, as well as by the increase in the training and match demands [61]. Despite being poorly adopted in youth soccer studies, this parameter has been widely used in rugby epidemiological studies [111–113], proving to be very useful to quantify the overall impact of an injury [103].

Of the total amount of articles, only nineteen reported injury incidence rates in female soccer players. The overall injury incidence ranged between 1.1 [40] and 7.20 [65], and also in this case the rate was higher during matches than during training [51, 57, 65, 92]. The overall range found was very similar compared to injury incidence in male players. In fact, several studies which investigated injury rates in young male and female soccer players [72, 78, 83] did not find significant differences, although other studies [51, 55, 76] reported a higher injury rate in the female sample. While it is well recognised that female athletes are more prone to ACL injury [114], due to hormonal, anthropometric and biomechanical factors, it is not yet clear whether sex difference may affect predisposition to other kinds of injury. Only a few studies [49, 57, 72, 78, 88, 92] reported injury severity in young female soccer players, and the use of different methods of classification makes any comparison difficult.

### Injury incidence according to participant level

The overall injury incidence identified in elite and non-elite male young soccer players was very similar. By contrast, the training injury rate observed was slightly higher in elite players. However, substantial differences were found comparing match injury rate. The range was from 2.84 [67] to 47.7 [43] in elite young soccer players, and from 1.8 [92] to 24.67 [65] in non-elite young soccer players. Although differences in injury risk between elite and non-elite groups have been investigated in adult soccer players [115], there is a lack of information in young soccer players. The higher injury rate found during training and, in particular, during matches may be explained by the higher play intensity that increases with the competitive level.

In female young soccer players, however, it is not possible to make any comparisons due to the scarcity of information in elite groups.

### Injury types and mechanism

Understanding the mechanisms underlying soccer-related injuries allows effective prevention strategies to be developed [116]. The most common types of injury reported in young male soccer players were muscle strains and ligament sprains [25, 36, 38, 43, 46, 64, 66, 67, 70, 89]. Other studies reported a higher prevalence of contusions/haematoma [42, 52, 69], which are more predominant during matches [21] or tournaments [41, 55], explained by the high intensity and speed required during these events. Fractures, lacerations and tendinosis appear to be less frequent in young soccer players [36, 42, 43, 56, 69, 79]. However, injury types may present different distribution and variability according to chronological and biological age. A higher proportion of muscle strains was observed in older players [22, 42, 62, 78] compared to younger ones. The increase in training load and competitiveness combined with incomplete muscle mass development may predispose athletes to muscle strains [96] and explain this finding. In addition, when injury types were analysed according to skeletal age, strains were more common in early maturer players [71]. In contrast, osteochondrosis disorders such as Osgood-Schlatter’s disease were mostly found in younger players, in particular at the beginning and at the end of their growth spurt [96]. In fact, the growth spurt phase represents a critical moment for young athletes, when bone and soft tissue development could lead to a reduction in flexibility and in turn to growth-related injuries. Contrarily, young female soccer players appear to be more prone to ligament sprains than muscle strains [41, 45, 49, 51, 72]. In this regard, Del Coso et al. [45] speculated that hormonal release during the different menstrual phases (i.e. progesterone, oestrogen) may affect ligament laxity and neuromuscular control. In support of this hypothesis, O’Kane et al. [117] reported a higher risk of injury in postmenarchal players. Contusions also had high prevalence in young female soccer players [41, 49, 78, 86], while fractures, lacerations, and growth-related injuries had a low impact [41, 45, 49, 86].

Less agreement was found regarding the mechanism of injuries in male youth players. Several studies recorded a higher rate of contact injuries [12, 21, 43, 51, 56, 70, 78], but at the same time many others reported a higher prevalence of non-contact injuries [18, 37, 39, 44, 47, 60, 70]. These results highlight the need to act bidirectionally in order to promote prevention strategies and reduce the risk of injury: first, developing good habits [78] and improving adherence to a fair-play policy in order to reduce violent behaviour linked to contact-related injuries [118]; on the other hand, trying to avoid non-contact injuries through monitoring the weekly training load [23], improving neuromuscular control [119], and promoting intervention strategies such as adoption of the “11+ Kids” programme [120].

In contrast, in female soccer, almost all the studies [24, 49, 51, 78, 87, 88] reported a strong prevalence of contact injuries, with the percentage ranging from 47% to 94%. This discrepancy with male soccer is not very clear. However, it is possible to speculate that different technical and tactical skills, as well as the adoption of different rules, may affect the results and the differences between the sexes.

### Anatomical location

Lower extremities were the most common injured body region in both male and female soccer players. However, it is possible to observe different anatomical distributions according to sex. In male soccer players the ankle and foot [12, 20, 23, 36, 42, 51, 65, 80, 96] together with the thigh [18, 39, 43, 48, 64, 67, 70] were the most common body parts injured. When reported, the anterior thigh showed higher prevalence compared to the posterior thigh [12, 36, 47, 54, 74, 80]. Regarding ankle injuries, the injury rate seems to increase with players’ age [20, 22, 35, 42]. One study [37], which focused exclusively on ankle injuries, confirmed this trend. According to previous studies [18, 20, 60], increasing volume exposure in training and matches, as well as increasing speed and muscle mass, may explain this tendency.

Furthermore, other studies recorded a high prevalence of groin injuries [39, 43, 48, 74, 80, 96]. In particular, one study found a higher rate of groin injuries in early maturer players [71], who, presenting an advanced biological age, were more predisposed to this kind of injury, as reported in a previous systematic review [121]. On the other hand, upper body and upper extremities showed a lower prevalence [12, 18, 36, 39, 41, 44, 47, 80] except for one study [38] conducted on goalkeepers, who, for reasons connected with playing position, are more exposed to elbow, forearm, wrist, and hand injuries. However, upper limb injuries might not be considered relevant to soccer participation, and therefore this could lead to their underestimation in epidemiological analysis [102].

In young female soccer players, the ankle and foot registered a high injury rate [35, 45, 49, 65, 72, 78, 85] as in male players, but they presented more knee injuries [41, 45, 49, 72, 88, 90]. As previously mentioned, this discrepancy could be explained by different sex characteristics: anatomical (Q angle), neuromuscular (hamstrings/quadriceps ratio), and hormonal (i.e. oestrogen, progesterone and relaxin) [122].

In female, as in male, young soccer players, upper body and upper extremities presented low prevalence [41, 45, 78, 90].

## LIMITATIONS OF THIS REVIEW

This review presents several strengths; to the best of our knowledge, this is the first review that combines epidemiological data and injury risk factors. Moreover, unlike previous reviews on young soccer players, female players were included in the analysis.

However, several limitations must be considered. The heterogeneity of the studies, mainly due to the different injury definitions used, did not allow us to perform statistical or meta-analysis of the results. Furthermore, the follow-up period was highly variable, ranging from a few days during tournaments [93] to 10 seasons [42, 71].

Moreover, studies reporting injuries in retrospective design were included. In this case, recall bias may underestimate the real number of injuries.

Several factors contributed to make comparison between articles difficult: some articles reported injury prevalence without injury incidence [18, 47], many authors reported injuries collected with different age ranges, the severity of injuries relied on contrasting classification methods [19, 23, 96] and, regarding anatomical location, many studies used different strategies to group the various anatomical districts.

Including articles regardless of playing level allows one to increase the amount of data to analyse; however, different technical levels, coaching style, and age of specialisation may affect injury incidence.

## CONCLUSIONS

Although soccer is generally considered a safe sport, injuries in young soccer players may have serious consequences: dropout, sequelae, and economic impact. The interest in the analysis of injuries in youth soccer has increased exponentially in the last years. The introduction of international consensus statements [98, 103] made it possible to increase the quality of the studies (injury definition, severity definition, types of injuries recorded). Moreover, there has been an increase in the number of players involved and in the length of studies. In addition, a comparison between the recent studies (last 2–3 years) with the past studies shows the increased use of parameters such as injury burden [61, 62] that allow better quantification of the injury impact in young soccer players.

Our analysis showed a different predisposition to injury according to sex, chronological and biological age. Injury incidence tends to increase with increasing age, and it is higher in matches and tournaments than in training. The growth spurt represents a period of high vulnerability in young male soccer players. However, further studies should clarify whether sex and maturity status have an impact on injury incidence. Male soccer players seem to be more prone to muscle strains and ligament sprains affecting particularly the ankle and thigh. Female players meanwhile suffer more ligament sprains located in the ankle and knee. Severe injuries are less frequent but tend to increase in older players. Therefore, the injury incidence may be high in young soccer players, but it depends on numerous factors including age, sex, and maturity. Knowledge of individual characteristics is needed in order to promote individualised prevention programmes.

Future studies should try to further investigate how the injury rate changes during the different development periods of young soccer players. Moreover, we observed a wide disparity between the studies conducted in male compared to female soccer. Therefore, considering the real absence of consistent investigation, future studies should focus more on epidemiological analysis of female soccer players.

## Funding

The authors received no specific funding for this work.

## Conflicts of interest/Competing interests

The authors have declared that no conflicts/competing interests exist.

## Contributorship

MM and AT was responsible for the conception and design of the study. MM, AT and AF conducted the literature review. MM, AT and MG contributed to data collection and interpretation. The article was written by MM and AT. All authors contributed to the reviewing of the manuscript.
